# Nurses' Experiences of the Caring Role during the COVID-19 Pandemic: A Scoping Review of Qualitative Research

**DOI:** 10.1155/2024/7147203

**Published:** 2024-08-23

**Authors:** Mary O' Regan-Hyde, Caroline Dalton-O Connor, Angela Flynn, Ashling Murphy, Vera J. C. McCarthy

**Affiliations:** University College Cork, College Road, Cork T12 K8AF, Ireland

## Abstract

**Aims:**

To synthesize the evidence on nurses' experiences of their caring role during the COVID-19 pandemic and identify emerging concepts that have affected nurses within the caring role in relation to (a) their professional lives and (b) their personal lives.

**Background:**

The concept of caring is central to the science and art of nursing practice, and fulfilment of the caring role is fundamental to the profession. The COVID-19 pandemic imposed unprecedented change globally transforming the caring role of the nurse. The WHO highlights that a well-supported workforce is paramount to emergency preparedness; therefore, understanding the experiences of the nurse's caring role during the COVID-19 crisis is paramount to practice in future healthcare crises.

**Methods:**

A scoping review. *Data Sources*. Studies published between January 2020 and November 2023 were identified from the following databases: Cumulative Index to Nursing and Allied Health Literature (CINAHL), Coronavirus Database, PUBMED, PsycINFO, PsycArticles, Scopus, Web of Science, and SocINDEX. *Reporting Method*. The scoping review adhered to the Joanna Briggs Institute (JBI) Preferred Reporting Items for Systematic Reviews and Meta-Analysis Extension for Scoping Review (PRISMA-ScR) checklist.

**Results:**

The search identified 1,347 studies, subsequent review of title and abstract, resulted in 117 full-text papers for further eligibility screening, with a total of 52 studies being included in the scoping review. Findings were grouped thematically using the Braun and Clarke (2006) approach. The five distinctive themes that emerged were (a) emotional turmoil, (b) erosion of care, (c) relationships and solidarity, (d) expansion of role, and (e) professional growth.

**Conclusion:**

During the COVID-19 pandemic, there was an evolutionary shift in the caring role of the nurse, on a trajectory from emotional turmoil to professional growth. The process followed a theoretical framework of transformative learning that could support nurses' capability and preparedness in their caring role for future inevitable extreme events and crisis in healthcare. *Implications for Nursing Management*. Mapping current knowledge of the unprecedented COVID-19 crisis from a nurse's professional and personal perspective purposefully aims to highlight gaps for future research, education, and policy and is paramount to emergency preparedness and a well-supported workforce in future healthcare crisis.

## 1. Introduction

The concept of caring is central to the science and art of nursing practice and can be broadly defined as the interpersonal interactions between the nurse and the patient [1]. Within the professional role, it is the responsibility of the nurse to provide high-quality nursing care that improves patient outcome, well-being, and health [2]. In crises, nurses want to fulfil their professional role [3]; however, modification in care delivery can create new and unfamiliar tensions in the dimensions of caring between nurses, patients, and their families [4]. Impacts of the COVID-19 pandemic imposed unprecedented changes globally on an already overburdened healthcare system, in particular, on nurses in their caring role [5–7].

COVID-19,the acute and severe respiratory syndrome caused by coronavirus 2 (SARS-Co-2), was first detected in December 2019 [8] and spread rapidly worldwide causing multiple fears, risks, and ethical dilemmas for nurses, both professionally and personally. The exposure to trauma had many negative effects including exhaustion and burnout, posttraumatic stress disorder (PTSD), and mental health issues such as anxiety and depression [5, 9, 10]. The pandemic also compounded preexisting inequalities in social, economic, and political systems, exposing vulnerabilities associated with age, race, social status, and in particular, gender, equality, and women's rights [11–14]. The healthcare workforce worldwide, consisting of over 67.2% women with twenty-eight million nurses in healthcare delivery, meant that during the COVID-19 pandemic, there was a disproportionate health and well-being risk felt by women working in healthcare, with nurses experiencing increased caregiving responsibilities, decreased leadership opportunities, reduced access to safety resources, and increased rates of ill health [15–17].

Although the COVID-19 crisis stigmatized nurses and inflicted risks and anxieties, it also created an awareness of the importance of caring responsibilities and generated professional and ethical growth in the caring role [18]. Teamwork was a vital element in the chaotic environment of the crisis, and unified goals, as well as collegial support and solidarity, evoked a shift from the healthcare team to healthcare family [19, 20]. The caring role of the nurse extended during the pandemic with increased learning in the clinical field [21], and new perspectives on nurses' personal health and well-being [22]. The expanded nursing role improved not only nurses' altruism and satisfaction but also their confidence and ability to deliver safe patient care during the unprecedented event of the COVID-19 pandemic [23, 24].

COVID-19 exposed many challenges within nursing such as inequity, financial austerity, inadequate managerialism, and governmental support, and while nurses throughout the pandemic were required to implement health policy, they rarely got to inform policy, with the nursing voice unrepresented at a public or government level [17, 25]. The WHO prioritises health in terms of the reduction of inequity and highlights that a qualified, motivated, and well-supported workforce is paramount to the development of people-centred healthcare systems and emergency preparedness [26]. The opportunity to question behaviours and translate experiential knowledge secondary to extraordinary circumstances could transform and improve outcomes in future inevitable crisis in healthcare [17, 27]. Hence, a review that focuses on the experiences of the nurses' caring role during the unprecedented and extraordinary circumstances of the COVID-19 crisis is required, encompassing the capabilities and vulnerabilities of the nursing profession, to inform education, policy, research, and practice in future healthcare crises.

### 1.1. Aim

The aim of this scoping review is to synthesize the qualitative evidence on nurses' experiences of their caring role during the COVID-19 pandemic and identify emerging concepts that have affected nurses professionally and personally within the caring role. Mapping current knowledge of the unprecedented COVID-19 crisis from a nurse's perspective purposefully aims to highlight gaps for future practice and research.

## 2. Design

### 2.1. Design

A scoping review is the form of evidence synthesis chosen to address this research question and to facilitate systematic mapping of the emerging foundational concepts and identification of practice, policy, and knowledge gaps [28]. This review adhered to the Joanna Briggs Institute PRISMA-ScR (Preferred Reporting Items for Systematic Reviews and Meta-Analyses extension for Scoping Reviews) checklist, which ensures the methodological rigor in the conduct of the scoping review [29] and ensures transparency and reproducibility of the findings. The JBI mnemonic “PCC” (Population, Concept, and Context) is used to construct a meaningful and clear title that reflects the core elements of the research and constructs clear and meaningful objectives and eligibility criteria, demonstrating congruence between the title, aim, and inclusion criteria [29] (Table 1).

### 2.2. Search Methods

A broad search identifying relevant studies eligible for inclusion in the scoping review was conducted using predetermined criteria and search terms. Eight electronic health, behavioural, and social science focused databases including CINAHL, Coronavirus Database, PUBMED, PsycINFO, PsycArticles, Scopus, Web of Science, and SocINDEX were searched. Synonyms and search terms were selected from MeSH, index terms, and subject headings, in collaboration with librarian services. The Boolean terms “OR”/“AND” were used, and the search was carried out for the following terms: (nurse OR nurses OR nursing) AND (COVID-19 OR coronavirus OR 2019-n-cov OR cov-19 OR 2019 pandemic OR novel coronavirus) AND (experiences OR perceptions OR attitudes OR views OR feelings OR perspectives OR opinions) AND (caring). The database search for this scoping review was conducted from October 2022 to November 2023 for papers published between January 2020 and November 2023. Limiters were applied for each database search as per inclusion/exclusion criteria including published in English, date ranges, full text, and peer-reviewed journals (Table 2).

### 2.3. Eligibility Criteria

The multiple domains and clinical settings in nursing during the COVID-19 pandemic warranted specific and iterative policy and guidance according to speciality and escalating need according to the area. To understand the complexities and emerging phenomena arising from nurses' lived experiences during the COVID-19 pandemic, in this scoping review, studies using qualitative methodology, data collection, and analysis were included to capture the detailed voices of the participants in the acute hospital setting, as the most focused context in relation to caring responsibility and the COVID-19 crisis [30]. Empirical studies considered for the scoping review had the following inclusion criteria: (a) nurses, (b) caring for patients infected with COVID-19, (c) in the acute hospital setting, (d) qualitative studies, (e) published between January 2020 and November 2023, (f) peer-reviewed, and (g) published in English. The exclusion criteria included unqualified nurses, nurses working with non-COVID-19 patients, nurses working in the nonacute hospital setting, nonqualitative, non-peer-reviewed, and non-English studies.

### 2.4. Critical Appraisal

As this scoping review is an overview of the nature and diversity of the current evidence and knowledge available, a critical appraisal of methodological limitations was deemed unwarranted [31], as findings are limited to guidance on policy making or as part of further empirical research [29].

### 2.5. Data Extraction and Synthesis

Data were extracted from the included studies by the first author (MORH), according to the PRISMA-ScR checklist [32]. Data were recorded using a predefined data extraction table, determined by the variables relevant to the review. Data were extracted under the following headings: author(s), year and country, study aim, study population, study design and data analysis, and key findings relating to the review questions. Extracted data were checked by VMC, and inconsistencies were iteratively discussed and resolved between MORH and VMC (Table 3). There were no unresolved issues to be reviewed by a third author. Data were synthesized according to the aims of the review [32] and following the Braun and Clarke process of thematic analysis [33]. Analysis of the data included familiarization, coding, ordering, categorizing, and summarizing data from the primary sources [33].

## 3. Results

**Figure 1:**
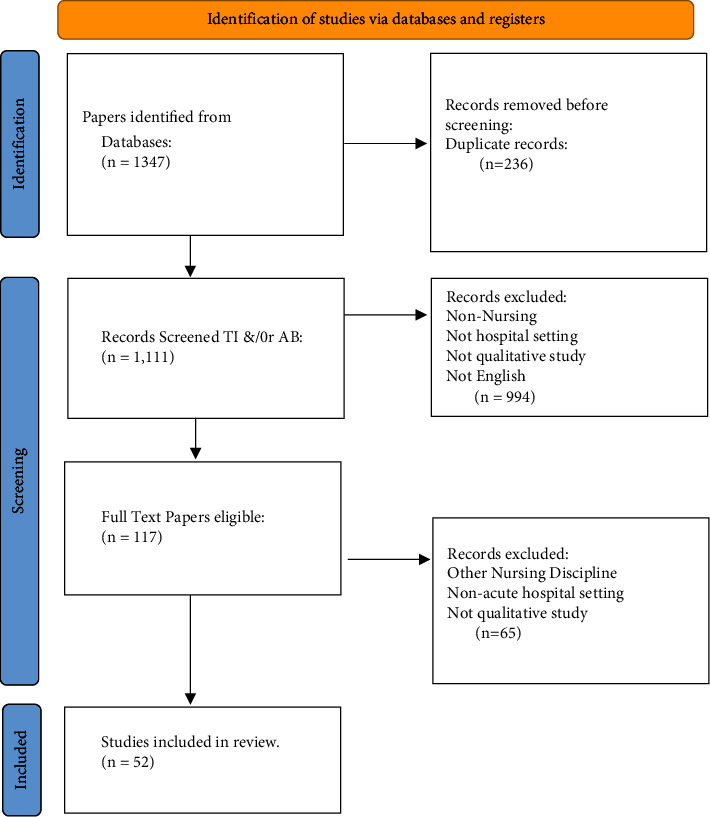
Preferred Reporting Items for Systematic Reviews and Meta-Analysis (PRISMA) flowchart.

The initial search of articles was independently conducted by MOR (first author). A total of 1347 studies were identified from the database searches with 236 duplicates removed, and papers were screened by title and/or abstract: PUBMED (*n* *=* 328), CINAHL (*n* *=* 271), Web of Science (*n* *=* 264), Coronavirus Database (*n* *=* 108), Scopus (*n* *=* 89), SocINDEX (*n* *=* 31), PsycINFO (*n* *=* 18), and PsycArticles (*n* *=* 2). Following the removal of nonapplicable records according to inclusion criteria (*n* *=* 994: nonnursing, non-peer-reviewed, and not acute setting), 117 full-text papers were identified for further screening. The papers were stored in the Zotero reference management system repository [34] and uploaded to Covidence, a collaborative systematic review management system [35]. Of these, 65 articles were excluded and the remaining 52 studies were considered eligible for the scoping review. A PRISMA flowchart outlines the findings (Figure 1).

### 3.1. Characteristics of the Included Studies

**Figure 2:**
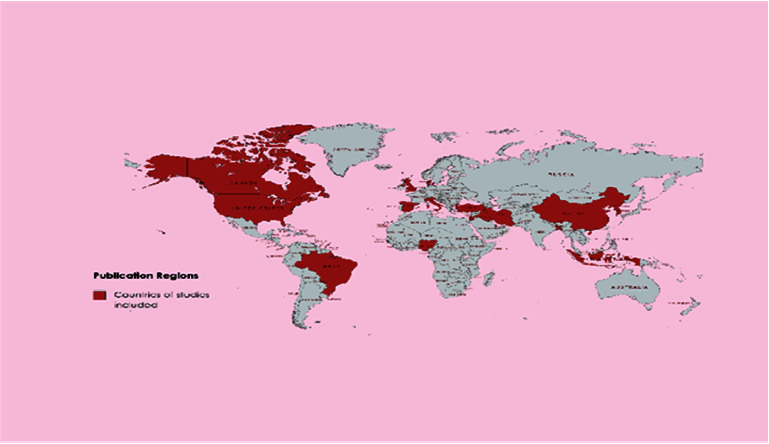
Geographical range of included studies.

There were 52 original peer-reviewed articles included in this scoping review. Most of the papers were published from May to December 2022. The studies encompassed a wide geographical range, of 20 different countries: Iran (*n* = 12), Turkey (*n* = 9), USA (*n* = 8), China (*n* = 5), Spain (*n* = 2), South Korea (*n* = 2), and one from each of the following countries Bangladesh, Brazil, Canada, Denmark, Indonesia, Iraq, Israel, Italy, Jordan, Korea, Nigeria, Pakistan, Qatar, and UK (Figure 2). The study design in all the articles was purposefully qualitative to provide a detailed primary perspective of the nurse's voices captured in context. Methodological approaches in the research studies included descriptive research (*n* = 30) and phenomenology (*n* = 22). Methods of data collection included semistructured interviews (*n* = 50) and journaling (*n* = 2). Due to the infectious nature of COVID, most interviews were via telephone (*n* = 15) and video link (*n* = 17), with some face-to-face interviews, where infection control measures were noted (*n* = 18), and journal entries (*n* = 2). Data analysis in the included studies was conducted either thematically (*n* = 13) or using content analysis (*n* = 39). Population size of nurses interviewed ranged from *n* = 14 to *n* = 46 and was purposefully at microlevel to capture the experiences of nurses in the acute hospital settings, including COVID-19 units, intensive care units, and hospital wards. In total, the experiences of 957 nurses were recorded from the studies in the scoping review.

### 3.2. Findings/Themes

The results of this scoping review are reported narratively under the five distinctive themes that emerged. These themes were (a) emotional turmoil, (b) erosion of care, (c) relationships and solidarity, (d) expansion of role, and (e) professional growth.

#### 3.2.1. Theme 1: Emotional Distress and Turmoil

The effects of emotional distress on the caring role of the nurse during the COVID-19 pandemic are clear in the literature and are referred to as a burden, toll, and psychological turmoil that paralysed optimum performance [36–38]. The included studies identified numerous emotions experienced by nurses during the pandemic including denial [39], grief [40, 41], sadness [20, 42, 43], loneliness [44], and anger [43, 45], which left nurses feeling overwhelmed and discontented [46–48]. Fear was the predominant emotion experienced in the included studies among nurses, both professionally [19, 21–24, 39, 40, 42–45, 48–64] and personally [18–23, 42, 43, 45, 47–49, 53, 54, 56, 59, 61, 64–68], threatening their well-being and family life [42, 69]. Fear of the unknown and uncertainty [3, 19–21, 38, 45, 56, 63, 70], and the infectious, novel nature and longevity of the disease powered that fear [46, 49, 50]. Fear of infection [19, 22, 50–52, 55, 61] triggered associated stigma, and nurses were branded as “coronurses” because of their caring role, and in the studies, they refer to their guilt and shame [55, 71]. The ease and rapid spread of the disease caused a major fear of transmission [43, 58, 59], especially when it traversed to family life [18, 23, 36, 37, 42, 43, 45, 47, 49, 53, 54, 62, 64, 65, 68], resulting in significant stress and anxiety for nurses in their role as carer at work and in the home [18, 23]. The fear of death was regularly referred to in the articles [19–22, 40, 45, 46, 48, 55, 57, 64, 66, 68, 72, 73] and was compounded by media coverage of death tolls, deaths of healthcare workers, and death scenes causing immense psychological pressure for nurses [22, 55, 57]. Fear affected nurses' attention, understanding and decision-making ability, and nurses' recounted elevated levels of anxiety and fear during the pandemic which hindered performance in caring [74]. Professional commitment is also cited as creating ethical and moral dilemmas, having to decide between patients and family responsibilities; however, nurses still immersed themselves in patient care and managed their anxiety and pressures in the volatile environment [19].

#### 3.2.2. Theme 2: Erosion of the Caring Role

The COVID-19 pandemic eroded the caring role with limited therapeutic time, isolation, and inadequate resources [40, 51]. Although a necessary infection control measure in the management of the virus, there is regular reference made in the studies regarding the significant effects of isolation nursing on the caring role of the nurse [40, 42, 48, 52, 57, 58, 60, 61, 68, 71, 73], particularly, in relation to clustering of care [61, 68, 71]. There was restricted human touch and less direct communication in patient care [68, 71]. Inadequate resources led to risk of exposure to the virus, placing nurses in unfavourable conditions [39, 48, 55, 72]. Nonstandard, poor quality, and inaccessible personal protective equipment (PPE) caused physical discomfort that affected nurses and complicated patient care [43, 50, 51, 56, 59, 63, 75, 76]. Time limitations, increased workload, and patient instability created an involuntary tension state for nurses [40, 58]. Many of the nurses' narratives in the papers alluded to the nurses' being drained of energy, with lack of breaks impacting concentration and lucidity [21, 36, 58, 60, 73, 75, 77]. Other cited working conditions that impacted nurses' safe delivery of care include unfair work distribution, redeployment, and new working environments [50, 56, 69, 72, 75, 78].

Professional knowledge deficits in the management of the disease [46, 52, 66], iterative policies and guidelines [19, 41, 64, 79], and insufficient training and media-driven knowledge are described by nurses in the studies as frustrating [37, 57, 58, 68]. The rapid transformation of clinical tasks [9, 47] and the severity and complexity of the disease made nurses anxious, hindering the delivery and quality of care [18, 45, 75]. Caring is the unifying force for nursing [80], and the enforced disturbance of COVID-19 caused several ethical and moral dilemmas that led to nurses feeling insecure in the role [67]. Professional dilemmas around responsibility for their own health and compassion towards patients were described as a constant challenge [22, 79]. The fear and guilt of undertaking patient care [67] dictated the isolation and separation of nurses from their role as family carer and affected family well-being [59]. The unattainment of goals left nurses questioning their professional competencies and the scope of their caring role amid the pandemic [47] as well as their autonomy and professional values in care [59].

#### 3.2.3. Theme 3: Relationships and Cohesion

Multiple relationships were affected by the pandemic in the nurses' caring role, including interprofessional, patient, institutional, and personal relationships, as well as relationships with the media and the larger community. Evidence from included studies reveals that unified goals enhanced teamwork and interprofessional cooperation and evoked a shift from the healthcare team to healthcare family [19, 20]. Teamwork, a vital element in the chaotic environment of a crisis, was significant during the pandemic, and team relationships developed in the shortest and most difficult of situations, with new and prominent roles for nurses in a multidisciplinary team approach [22, 54]. Collegial solidarity was one of the important elements mentioned by nurses in their professional role during the pandemic, giving them support, optimism, and hope [3, 40, 61, 63, 79]. Connection to coworkers helped foster feelings of mental well-being, counter anxiety, and enhance positive patient care and outcomes [41, 60]. Support and collaboration of colleagues assisted adaptation, helping associates remain committed to caring and was reflected among nurses by continuously “showing up” for their colleagues, as well as their patients [22, 41]. Conversely, the literature also highlights difficulties in working with different team members, insufficient communication, and conflicts, and performance competition between teams is also cited as decreasing the quality of care [44, 55, 72].

The behaviour, cooperation, and compassion of patients for nurses in their professional caring were emotive and motivating, and positive response from patients supported the importance of the nurse's caring, strengthening the nurse-patient relationship [22, 60, 64]. However, verbal abuse and aggressive behaviour from patients and relatives during the COVID-19 crisis, in some instances, impacted negatively on nurses [51, 73], and nurses relationships with organisational structures ranged from positive to disheartening [19]. Insufficient support by authorities, with unrealistic expectations and staffing shortages, caused collective frustration for nurses [18]. Institutional relations were fraught with inequality during the pandemic in relation to inadequate salaries, benefits, leaves, and the type of contracts for nurses [23].

COVID-19 pervaded nurses' personal lives and affected their caring role in family and social responsibilities [67]. Stress caused by the disease affected nurses physically, psychologically, and emotionally, and some studies revealed that it instigated domestic upset and hostility [46, 67, 73]. Disruption of life plans such as family celebrations and travel plans and more personal bonds such as breastfeeding or sentimental moments as a child taking their first steps were lost according to nurses interviewed in the papers [49, 51]. Family relationships were a priority during the pandemic for nurses and the fear of transmitting COVID-19, particularly to elderly relatives and children in their personal caring roles, gave rise to changes in personal lifestyle routines, self-imposed isolation, and quarantine away from family and loved ones [3, 22, 39, 44, 49, 66]. Nurses expressed that they had limited time to deal with things outside of work and felt burnt out attempting to provide reciprocal communication between family life and work [44, 65]. Additional responsibilities in nurses' personal caring role unfolded in the pandemic with homeschooling, affecting parental roles in the family, and difficulties in education at home for some children [52, 73].

Nurses faced altered reactions from society, being stigmatized as carriers of the disease, and sometimes alienated as nurses [22, 49, 65], while external recognition by media and other organisations improved the image of nursing and pride in the professional role and general appreciation. This increased nurses' motivation to work during the COVID-19 crisis [21, 43] however contrary to that, some studies identified that nurses also witnessed social panic due to unrealistic reporting, termed as an infodemic, causing nurses' increased stress and anxiety in caring role [20, 23, 40, 81].

#### 3.2.4. Theme 4: Expansion/Evolution

The role of the nurse extended during the pandemic and according to many studies was an opportunity to learn new skills, increasing theoretical and practical knowledge particularly in relation to infection control and isolation nursing [45, 48, 79, 82] helping them fulfil professional responsibilities [61]. Nurses took on multiple new roles such as conduit for family members, advocate, and guarantor for isolated and unconscious patients [21]. The role of therapeutic communication, calming patients' loneliness with true active listening and paying close attention to imperceptible sound, being a central messenger between families and patients [22, 55, 57] and counselling patients during the pandemic were all essential functions of the nurse [18, 55, 73]. The function of the nurse as proxy or surrogate family to patients in the absence of their own relatives due to infection control regulation formed part of the extended caring role of the pandemic nurse [52]. Psychological care encompassed patients and their families and was critical to holistic care delivery [69]. Relatives were reliant on nurses for information and support, and relationships were mixed with gratitude and frustration [20]. Technology and electronic devices such as cell phones and tablets for patient-family intercommunication assisted nurses in bringing patients and families together, particularly at sensitive and vulnerable times such as breaking bad news and end-of-life discussions and goodbyes [20, 62, 66, 71]. According to nurses in some of the articles, the experience of death characterised uniquely by the pandemic [20, 21, 52, 56] and managing death and loss was overwhelming and emotionally exhausting [46, 64, 81] and not experienced previously during nurses' professional careers [41].

The studies also highlighted that nurses took on a new perspective to their personal health, extending caring to their own lives [22]. New rituals were performed, disinfecting themselves before entering their homes, protecting their families from the virus [65, 68, 81]. Nurses made positive lifestyle changes in the form of physical exercise, relaxation through reading or meditation, keeping a diary, and spirituality [20, 44, 52, 54, 64, 83]. Coping strategies and distraction helped normalise the care of families and nurses reflected on a more positive daily attitude, spending more time with children and planning their future [42, 72]. In their personal lives, nurses found that the support of colleagues and family made them more resilient and better able to perform in their lives professionally and personally [46, 62].

#### 3.2.5. Theme 5: Professional Growth

The evidence suggests that realisation of the importance of the nurses' caring role has inspired professional growth in nursing [48]. A rebirth of Nightingale's philosophy of advocacy and commitment to the patient was evident from the literature in relation to altruism and endorsement of new professional responsibility in a humanitarian role, as opposed to obligation or resignation [22, 64, 68, 84]. Expansion of professionalism increased self-esteem and pride, affirming career choice for some, engendering a sense of accomplishment [36, 45, 46, 56, 84]. There was a feeling of achievement when patients recovered in the pandemic that amplified motivation, satisfaction, and pride in the nurses' role. Positive professional identity motivated nurses in their caring role, valuing the growth in their responsibility and self-worth [73, 85].

While studies revealed that nurses' knowledge and skills in epidemic nursing, rescue tasks, and infection control were strengthened, it also increased nurses' ability to cope in future similar events and a readiness for managing infectious diseases and future healthcare crises [55, 57, 58, 60]. According to the nurses in one study, being novices in the new COVID-19 wards was threatening but also strengthened nurses professionally and personally demonstrating ability to manage extreme situations [50]. With new knowledge comes increased scope, autonomy, and an appreciation for the importance of evidence-based informed care and nurses in many of the studies during COVID-19 demonstrated the capability and innovation of combining intuition with critical thinking skills in clinical practice [20, 46, 77]. The expanded nursing role improved not only nurses' altruism and satisfaction but also their confidence and solidarity, in the ability to deliver safe patient care in an iterative caring role during the pandemic augmented the capabilities of nurses for future crisis in healthcare [23, 24].

## 4. Discussion

The key findings from this study, in the context of nurse's experiences in the acute hospital setting, within the concept of their caring role, both professionally and personally, emerged as five distinctive themes. These themes followed a pattern of transformative learning for nurses that was challenging, emotive, and rewarding. They include (a) emotional turmoil, (b) erosion of care, (c) relationships and solidarity, (d) expansion of role, and (e) professional growth. Reflecting on the facilitators and barriers in the caring role of the nurse during the pandemic could support new nursing autonomy and capability, professionally and personally, framing an organised pathway in the caring role for future healthcare transitions.

The challenges experienced by the nurses in their caring role were iterative and ranged from fear and anxiety because of the sudden, novel, and highly contagious nature of the disease, to new growth in relation to knowledge and skill acquisition and increased professional capability. The evidence suggests that the transition was a complex journey of emotional turmoil, intense and high-risk working environments, and relational interdependencies that affected nurses physically, psychologically, and psychosocially [18, 45, 75]. Nurses were faced with new dilemmas within the caring role in relation to personal safety, managing insufficient resources, reduced patient contact, and unique death experiences [22, 55, 57]. The impact of the professional caring role on personal family life and risk of COVID-19 transmission, especially to children and elderly relatives caused the most anxiety. Nurses and their families were also subjected to social stigmatization being excluded from colleagues, family, and community events [60]. Amid the adversities however, nurses adapted and demonstrated transitionary learning, developing strategies and capabilities that reduced the risk of contagion while managing the caring role in their professional and personal lives.

There is an abundance of evidence on nurses' caring experiences during the COVID-19 pandemic in relation to emotional fear and anxiety [86, 87], professional nursing values and growth [86–88], and confronting difficulties in both career and family life [87, 89] that validate the findings of this review. In addition, the transformative learning evident from the findings is a further construct and an important factor in the transitionary process of the nurse's journey during the COVID-19 crisis. Transformative learning is complex and a process around which experiences are understood by formalizing and reflecting on a problem as defined by Mezirow's theory [90]. The basic premise is that humans formulate views based on assumptions of negative and positive experiences which influence future learning. Transformative learning affects the learned experience by creating a paradigm shift, consequently altering future experiences [91–93]. This transformative progression requires learners to become more autonomous and capable of determining their own actions in future situations [93]. Nurses in their caring capacity both professionally and personally unconsciously experienced transformative learning throughout the pandemic which followed the Mezirow methodological theory in a ten-step approach [94] (Table 4).

Their experiences highlight multiple dilemmas in the caring role in relation to resources [68, 75], ethical decisions [39, 49, 59], and restrictions to caring within their personal lives [18, 81]. Nurses experienced inefficiencies and substandard performance [18, 45, 75] which in turn led to feelings of guilt or shame [55, 71] and questioning of their caring role [47]. Expansion of the caring role, professionally [21] and personally [46], increased acquisition of knowledge [45, 48, 79, 82] and formation of solidarity in relationships [3, 40, 61, 63, 79], enhancing the nurses' caring role during the pandemic. However, knowledge and time constraints [46, 52, 66] and iterative policies and guidelines and lack of institutional support [19, 41, 64, 79], as well as rapid transformation of clinical tasks [49, 55], afforded nurses no time to reflect on their caring role and plan a course of action during COVID-19. Learning in the clinical setting [21], behavioural modifications in family life [56], and self-care strategies [20, 44, 52, 54, 64, 83] were developed as safety and preservation techniques rather than a formulated construction of competence and self-confidence [90]. The WHO highlights that a qualified, motivated, and well-supported workforce is paramount to the advancement of people-centred healthcare organisations, and emergency preparedness [26] and the introduction of interventions can inform the psychological trauma or growth of a workforce [95, 96]. It is an opportune time for nurses to reflect on their experiences and develop the transformative learning gained during COVID-19 to build on the sustainability of the caring role of the nurse both professionally and personally and generate new perspectives for future adverse healthcare events [90].

## 5. Conclusion

This scoping review explores relevant qualitative evidence-based papers on the experiences of nurses in their caring role during the COVID-19 pandemic. As the COVID-19 pandemic was recent, and knowledge of the virus and nursing care required was novel and iterative in nature, the critical comparison of the evidence has revealed that during the pandemic, there was an evolutionary shift in the caring role of the nurse, on a trajectory from emotional turmoil to professional growth. Although not definitive, the pathway followed a pattern of transformative learning for nurses that was challenging, emotive, and rewarding. Reflecting on the facilitators and barriers in the caring role of the nurse during the pandemic could support new nursing autonomy and capability, professionally and personally, framing an organised pathway in the caring role for future healthcare transitions.

## 6. Strengths/Limitations

Strengths in the design of this review include studies from a wide geographical range and adherence to the PRISMA extension of scoping reviews' framework [29], a replicable guideline in relation to reporting of the sourcing, selection, extraction, and analysis. The application of findings to Mezirow's theory and framework for translational learning [93] facilitates identification of a pathway for further advances in research gaps and consideration for policy and learning for nurses in crisis awareness/preparedness in the caring role.

This study is limited in relation to the inclusion of articles written in the English language although the pandemic was a worldwide crisis. The study aimed at understanding the complexities and emerging phenomena arising from nurses' lived experiences during the COVID-19 pandemic. Studies using qualitative methodology, data collection, and data analysis were included to capture the detailed voices of the participants in context and provide consistency in data. The specific inclusion criterion of qualitative peer-reviewed studies for this scoping review therefore dictated exclusion of nonqualitative and grey literature. There is also an abbreviated time of three years (2020–2023), on the included articles. This, however, could equally validate the findings as experiences recorded were, for many of the included papers, within the COVID-19 period classified as a pandemic. The aim of this scoping review was to present an overview of the evidence on nurses' experiences of the caring role during COVID-19. As it was an overview of published studies, an examination of any methodological limitations and potential risk of bias of included studies was not conducted as outlined; however, conducting a quality appraisal may have identified some limitations in the included studies [97].

## Figures and Tables

**Table 1:** JBI (2015) PCC framework.

PCC element	Definition
Population	Nurses
Concept	Caring role
Context	Patients with COVID-19 virus in an acute hospital setting

**Table 2:** Search strategy.

Database (*n*)	Keywords and limits

PubMed (*n* = 328)	(((nurse [Title/Abstract] OR nurses [Title/Abstract] OR nursing [Title/Abstract]) AND (COVID-19 [Title/Abstract] OR coronavirus [Title/Abstract] OR 2019-n-cov [Title/Abstract] OR cov-19 [Title/Abstract] OR 2019 pandemic [Title/Abstract] OR novel coronavirus [Title/Abstract])) AND (experiences [Title/Abstract] OR perceptions [Title/Abstract] OR attitudes [Title/Abstract] OR views [Title/Abstract] OR feelings [Title/Abstract] OR perspectives [Title/Abstract] OR opinions [Title/Abstract])) AND (caring [Title/Abstract])
	Limiters/filters: full text, English, from 2020/1 to 2023/11

CINAHL Plus (*n* = 271)	AB (nurse OR nurses OR nursing) AND AB (COVID-19 OR coronavirus OR 2019-n-cov OR cov-19 OR 2019 pandemic OR novel coronavirus) AND AB (experiences OR perceptions OR attitudes OR views OR feelings OR perspectives OR opinions) AND AB (caring)
	Limiters/filters: full text, English, from 2020/1 to 2023/11, peer reviewed

Web of Science (*n* = 264)	(((AB = (nurse OR nurses OR nursing)) AND AB = (COVID-19 OR coronavirus OR 2019-n-cov OR cov-19 OR 2019 pandemic OR novel coronavirus)) AND AB = (experiences OR perceptions OR attitudes OR views OR feelings OR perspectives OR opinions)) AND AB = (caring)
	Limiters/filters: English, from 2020/01/01 to 2023/11/30, peer reviewed article, nursing

Coronavirus Research Database (*n* = 108)	Abstract (nurse OR nurses OR nursing) AND abstract (COVID-19 OR coronavirus OR 2019-n-cov OR cov-19 OR 2019 pandemic OR novel coronavirus) AND abstract (experiences OR perceptions OR attitudes OR views OR feelings OR perspectives OR opinions) AND abstract (caring)
	Limiters/filters: full text, English, from 2020/1 to 2023/11, peer reviewed

Scopus (*n* = 89)	ABS (nurse OR nurses OR nursing) AND ABS (covid-19 OR coronavirus OR 2019-n-cov OR cov-19 OR 2019 pandemic OR novel AND coronavirus) AND ABS (experiences OR perceptions OR attitudes OR views OR feelings OR perspectives OR opinions) AND ABS (caring)
	Limiters/filters: full text, English, from 2020/1 to 2023/11

SocINDEX (*n* = 31)	AB (nurse OR nurses OR nursing) AND AB (COVID-19 OR coronavirus OR 2019-n-cov OR cov-19 OR 2019 pandemic OR novel coronavirus) AND TX (experiences OR perceptions OR attitudes OR views OR feelings OR perspectives OR opinions) AND TX (caring)
	Limiters/filters: full text, English, from 2020/1 to 2023/11

APA PsycINFO (*n* = 18)	AB (nurse OR nurses OR nursing) AND AB (COVID-19 OR coronavirus OR 2019-n-cov OR cov-19 OR 2019 pandemic OR novel coronavirus) AND AB (experiences OR perceptions OR attitudes OR views OR feelings OR perspectives OR opinions) AND AB (caring)
	Limiters/filters: full text, English, from 2020/1 to 2023/11, peer reviewed

APA PsycArticles (*n* = 2)	(Nurse OR nurses OR nursing) AND (COVID-19 OR coronavirus OR 2019-n-cov OR cov-19 OR 2019 pandemic OR novel coronavirus) AND (experiences OR perceptions OR attitudes OR views OR feelings OR perspectives OR opinions) AND (caring)
	Limiters/filters: full text, English, from 2020/1 to 2023/11, peer reviewed

**Table 3:** Study characteristics.

Author, year, country	Research aim/objectives	Sample and setting	Research/methods and design	Data collection and analysis	Key findings: nurses' experiences of professional caring role in COVID-19	Key findings: nurses' experiences of personal caring role in COVID-19
Abdulah et al. 2022 [74], Iraq	The experiences of nurses in COVID-19 hospitals	Nurses (*n* = 12)	Descriptive research study	Semistructured telephone interviews Thematic analysis	(i) Fear of infection and stress/anxiety	(i) Fear for family
					(ii) Aggressive behaviour	(ii) Separation family/home
					(iii) Isolation-limited patient/colleague engagement	(iii) Social exclusion family/friends

Afzal et al. 2023 [62], Pakistan	Lived experiences of nurses caring for COVID-19 patients	Nurses (*n* = 30)	Descriptive research study	Semistructured face-to-face interviews Thematic analysis	(i) Mental, physical, and social health impacts (ii) Fear, anxiety, and depression (iii) Professional requirements/pride and humanitarian/motivation (iv) Cooperative colleagues	(i) Social response/family/society support (ii) Concern for family health

Ahmadidarreh-sima et al. 2022 [58], Iran	The care experiences of Iranian nurses during the COVID-19 outbreak	Nurses (*n* = 10)	Descriptive research study	Semistructured face-to-face interviews Content analysis	(i) Fear of transmission: anxiety/irritability (ii) Constraints: resources/workload (iii) High mortality and compassion fatigue (iv) Low patient engagement (v) Crisis preparedness	(i) Social exclusion (ii) Family support (iii) Separation (iv) Self-care

Akkus et al. 2022, Turkey	The experiences and challenges faced by nurses working in COVID-19	Nurses (*n* = 19)	Inductive research study	Semistructured telephone WhatsApp interviews Thematic analysis method	(i) Emotional fluctuations (ii) Clinical discourse and fear (iii) Patient relationships (iv) Uncertainty resource and information (v) High mortality and compassion fatigue (vi) Professional satisfaction/pride (vii) Unified goals healthcare team/healthcare family	(i) Social exclusion (ii) Fear (iii) Separation (iv) Concern (v) Self-care

Al-Amer et al. 2022 [59], Jordan	The lived experience of nurses caring for patients with COVID-19	Nurses (*n* = 10)	Descriptive phenomenological research study	Semistructured telephone interviews Data analysis Colaizzi's phenomenological method	(i) Fear and risk of transmission (ii) Ineffectual patient care (iii) Altered work environment (iv) Physical and psychological burden (v) Ethical dilemma	(i) Transmission to family (ii) Altered social relations

Arcadi et al. 2021 [21], Italy	Exploring the experience of nurses caring for COVID-19 patients	Nurses (*n* = 20)	Phenomenological hermeneutic research study	Semistructured video call interviews Data analysis Phenomenological hermeneutic approach	(i) Uncertainty and fear (ii) Skills inadequacy (iii) Altered workload (iv) Uniqueness of death experience (v) Role of conduit/advocate (vi) External recognition (vii) Professional cohesion/solidarity	(i) Anguish (ii) Guilt (iii) Separation (iv) Isolation

Broujeni et al. 2023 [76], Iran	Experiences of nurses providing care for patients infected with COVID-19	Nurses (*n* = 18)	Descriptive research study	Semistructured face-to-face interviews Content analysis	(i) Self-care for nurses (ii) Challenges in patient care/specific intervention/resource shortages/conflicting guidelines (iii) Communication with families/psychological support for patients	

Cerit and Uzum 2022 [79], Turkey	Determine experiences of nurses working in a COVID-19 clinic	Nurses (*n* = 9)	Phenomenological research study	Semistructured face-to-face interviews Content analysis	(i) Care provision/contagion (ii) Anxiety anger in caring role (iii) Resource constraints (iv) Team collaboration (v) Administrative difficulties and long working hours (vi) Changes in nursing practice (vii) Professional gains	(i) Fear (ii) Stigma

Chegini et al. 2021 [23], Iran	Nurses' experience in caring for patients infected by COVID-19	Nurses (*n* = 15)	Phenomenological research study	Semistructured telephone/face-to-face interviews Data analysis Colaizzi's phenomenological method	(i) Professional pride altruism solidarity (ii) Fear, stress, anxiety, obsession, and desolation (iii) Social panic false news (iv) Oranisational challenges (v) Discrimination	(i) Transmission to children

Conz et al. 2021 [45] Brazil	The experiences of ICU nurses caring for COVID-19 patients	Nurses (*n* = 20)	Phenomenological research study	Semistructured video call interviews Content analysis	(i) Uncertainty and fear (ii) Infection in colleagues (iii) Hindered care (iv) Death toll (v) Professional growth (vi) Leaving the profession	(i) Fear of transmission to family

Copel et al. 2022 [20], USA	Exploration of perceptions/experiences of nurses caring for patients with COVID-19	Nurses (*n* = 20)	Descriptive research study	Semistructured Zoom interviews Thematic analysis	(i) Uncertainty and role strain (ii) Variation in practice standards (iii) Unpredictability (iv) Managing death and loss (v) Teamwork/chaotic environment (vi) Relatives' reliance (vii) Electronic devices (viii) Cluster care difficulties (ix) Professional growth (x) Unrealistic media coverage	(i) Anxiety, fear, and sadness (ii) Emotional management (iii) Self-care (iv) Shared spaces (v) Social exclusion

Coşkun et al. 2021 [43], Turkey	Experiences and feelings of parent nurses who care for COVID-19 patients	Nurses (*n* = 26)	Descriptive research study	4 open-ended questions emailed for completion within a week Content analysis	(i) Insufficient resources	(i) Fear of transmission
					(ii) Fear of transmission and death	(ii) Separation anxieties
					(iii) General appreciation professional awareness	(iii) Two-way fear of death

Fernandez-Castillo et al. 2021 [40], Spain	To explore the experiences of nurses working with COVID-19 patients	Nurses (*n* = 17)	Qualitative research study	Semistructured interviews via video calls Thematic analysis	(i) Limitations of isolation nursing	
					(ii) Inexperience	
					(iii) Dehumanisation	
					(iv) Unholistic care/resources	
					(v) Fear, grief, and death	
					(vi) Infodemic/misinformation	
					(vii) Health team solidarity	
					(viii) Increased interdisciplinary and nurses' versatility	

Galadar et al. 2021, Iran	Nurses' perceptions of caring for patients with COVID-19	Nurses (*n* = 13)	Qualitative descriptive study	Semistructured in-depth telephone interviews Content analysis	(i) Care erosion (ii) Stress and anxiety (iii) Restriction resources (iv) Professional and ethical growth (v) Counselling of patients (vi) Insufficient support	(i) Dilemma/professional (ii) Fear and anxiety of transmission (iii) Separation from children

Gordon et al. 2021 [52], USA	To explore the experiences of critical care nurses caring for COVID-19 patients	Nurses (*n* = 11)	Descriptive research study	Semistructured interviews face-to-face and Zoom platform Content analysis	(i) Fear anxiety/stress (ii) Contracting/transmission (iii) Knowledge and skill deficit (iv) Isolation nursing (v) Patient empathy/nurse as surrogate family (vi) Physical symptoms PPE and work overload (vii) Disease complexity/death (viii) Support of coworkers	(i) Transmission to family (ii) Social stigma (iii) Healthcare hero perception (iv) Isolation/loneliness (v) Additional responsibilities/homeschooling (vi) Family support (vii) Self-care

Heydarikhayat et al. 2022 [81], Iran	To explore the lived experiences of nurses in caring with COVID-19 patients	Nurses (*n* = 13)	Descriptive phenomenological research study	Semistructured face-to-face interviews Data analysis Colaizzi's phenomenological method	(i) Whirlpool of stress	(i) Dilemma self-protection caring (ii) Protection of families
					(ii) Media	
					(iii) Mortality rates	
					(iv) Novel virus	
					(v) Essence/sacrifice of nurses	
					(vi) Unity team	
					(vii) Crisis aware	
					(viii) Spiritual connection	

Irandoost et al. 2022 [73], Iran	The problems and adaptation techniques of nurses caring for COVID-19 patients	Nurses (*n* = 16)	Qualitative research study	Semistructured interviews face-to-face and on the phone Content analysis	(i) High work pressure	(i) Disconnection (ii) Homesickness (iii) Role reduction (iv) Distant (v) Disruption in life plans
					(ii) Exclusion isolation	
					(iii) Patient care difficulties	
					(iv) Abuse aggression	
					(v) Death	
					(vi) Patient-family communication	
					(vii) Self-worth/growth in responsibility	

Jang et al. 2022 [60], Korea	Investigation of the meaning and essence of nurses' experiences of caring for COVID-19 patients	Nurses (*n* = 14)	Phenomenological research study	Semistructured face-to-face and online interviews Data Analysis Colaizzi's phenomenological method	(i) Fear and anxiety of novel virus	
					(ii) Isolation nursing	
					(iii) Substandard dignity in care	
					(iv) Burden of PPE	
					(v) Contrasting perceptions	
					(vi) Supportive colleagues	
					(vii) Nursing future readiness	

Jun and Rosemberg 2022 [63], USA	An exploration of experiences of frontline nurses in hospitals during COVID-19	Nurses (*n* = 22)	Descriptive research study	Semistructured individual phone interview Thematic analysis	(i) Fear/concern of unknown	(i) Social isolation (ii) Physical distancing at home
					(ii) Resource constraints	
					(iii) Peers and emotional support	
					(iv) Unrealistic expectations	
					(v) Powerful sense of duty	
					(vi) Pride in profession	
					(vii) Greater comradery	
					(viii) Changed in nursing practice	
					(ix) Diminishing trust/leaders	

Kackin et al. 2021 [72], Turkey	To determine experiences/psychosocial problems in nurses caring for COVID-19 patients	Nurses (*n* = 10)	Descriptive phenomenological research study	Semistructured interviews online Data analysis Colaizzi's phenomenological method	(i) Difficult working conditions	(i) Coping strategies (ii) Distraction normalisation (iii) Avoidance
					(ii) Unfairness	
					(iii) Decreased quality of care	
					(iv) Ethical decisions	
					(v) Management team members	
					(vi) Threatened/uncertainty	
					(vii) Witnessing death process	
					(viii) Social stigma	

Khanjarian and Sadat-Hoseini 2021 [39], Iran	Explanation of nurses' lived experiences providing altruistic patient care in COVID-19	Nurses (*n* = 25)	Interpretive phenomenology research study	Semistructured in-depth open interviews Thematic analysis	(i) Shock denial terror fear	(i) Dilemma staying/leaving (ii) Concern for family/health worker in family (iii) Family health
					(ii) Exposure	
					(iii) Challenging work conditions	
					(iv) Poor professional knowledge and experience	
					(v) Inadequate caregiving	
					(vi) Community's reaction	

Lee et al. 2022 [38], South Korea	What are nurses' experiences caring for COVID-19 patients	Nurses (*n* = 14)	Descriptive research study	Semistructured interviews on a virtual platform Thematic analysis	(i) Forced into uncertainty	
					(ii) Unfamiliar and insufficient performance	
					(iii) Emotional burden	
					(iv) Broadened scope	
					(v) Compensation deficit	

Levi and Moss 2022 [36], USA	Investigating the lived experiences of ICU nurses caring for COVID-19 patients	Nurses (*n* = 10)	Phenomenological research study	Semistructured telephone interviews Data analysis Colaizzi's phenomenological method	(i) Emotional toll	(i) Self-imposed isolation (ii) Fear of transmission (iii) Friends non-nursing lack of understanding
					(ii) Acuity of patients	
					(iii) Death (dying alone)	
					(iv) Lack of knowledge	
					(v) Substandard clustering care	
					(vi) Experiencing futility	
					(vii) Solidarity with coworkers	
					(viii) Relative/connections	
					(ix) Job satisfaction	

Liu et al. [13], 2020 China	To explore experiences of nurses combating COVID-19	Nurses (*n* = 15)	Descriptive research	Semistructured in-depth interviews face-to-face Thematic analysis	(i) Specialized nursing skills	
					(ii) Physically drained	
					(iii) Professional development	

Marey-Sarwan et al. 2022 [3], Israel	Nurses' experiences during the COVID-19 pandemic	Nurses (*n* = 18)	Descriptive research	Semistructured interviews face-to-face and Zoom Thematic content analysis	(i) Uncertainty/concern	(i) Social-family responsibilities (ii) Concern transmission (iii) Mental well-being
					(ii) Intensity of work	
					(iii) New roles	
					(iv) Professional development	
					(v) Peer support optimism and hope	

Mohammad and Lelievre 2022 [89], Canada	Gain insights into nurses' COVID-19 experiences	Nurses (*n* = 43)	Interpretive phenomenological approach	Semistructured interviews and online surveys Thematic content analysis	(i) Fear and anxiety/knowledge	(i) Transmission to family
					(ii) Personal safety secondary	
					(iii) Decreased morale	
					(iv) Stigmatization	
					(v) Social media coverage	
					(vi) Increased empathy	
					(vii) Improved team-nursing/tools	
					(viii) Isolation clustered care	
					(ix) Team dynamic of nurses/camaraderie	

Monjazebi et al. 2021 [53], Iran	An exploration of nurses' experiences of caring for COVID-19 patients	Nurses (*n* = 12)	Qualitative research study	Semistructured interviews and field notes Content analysis	(i) Security in caregiving	(i) Fear of transmission/family (ii) Young children or elderly parents (iii) Fear of contamination/living environment/community
					(ii) Fear of death	
					(iii) Confidence in skills	
					(iv) Diminished efficiency	
					(v) Mental exhaustion	
					(vi) Feeling conflicted	
					(vii) Reduced threshold of tolerance	

Moore et al. 2022 [68], USA	An exploration of the experience of US critical care nurses caring for patients with COVID-19	Nurses (*n* = 11)	Descriptive research study	Semistructured Zoom interviews Interpretative content analysis	(i) Lateral transmission	(i) Fear of transmission to family (ii) Socialization online with other nurses (iii) Rituals/disinfecting self and environment
					(ii) Breach in professional contract	
					(iii) Communication technology	
					(iv) Difficult conversation	
					(v) Siloed nursing care/isolation	
					(vi) Depersonalization of care	
					(vii) End-of-life care	
					(viii) Decision-making dilemmas	
					(ix) Moral distress	
					(x) Humanitarian purpose	

Moradi et al. 2021 [37], Iran	Exploring challenges of ICU nurses providing care for COVID-19 patients	Nurses (*n* = 17)	Descriptive research study	Semistructured face-to-face interviews Content analysis	(i) Psychological turmoil fear	(i) Transmission to family (ii) Aggressive/hostile to family (iii) Loss of peace in life (iv) Cessation of personal life (v) Domestic distress
					(ii) Aggression	
					(iii) Insecurity and ambiguity	
					(iv) Prolonged care	
					(v) Demotivation	
					(vi) Insufficient support	
					(viii) Lack of financial reimbursement	

Muz and Erdogan-Yuce 2021 [49], Turkey	Experiences of nurses caring for patients with COVID-19 at pandemic wards and ICU in Turkey	Nurses (*n* = 19)	Phenomenological hermeneutic research study	Semistructured Online video or audio calls Content analysis	(i) Fear of novel	(i) Transmission to family (ii) Alienated (iii) Isolated (iv) Separation
					(ii) Professional solidarity	
					(iii) Ethical care dilemmas	
					(iv) Societal support	
					(v) Professional growth	

Pariseault et al. 2022 [71], USA	Experiences of nurses caring for patients with COVID-19	Nurses (*n* = 17)	Descriptive research design	Semistructured interviews via Zoom Thematic analysis	(i) Communication barriers	
					(ii) Deprivation families/friends	
					(iii) Guilt/siloed care	
					(iv) Technology/devices	
					(v) End-of-life care	
					(vi) Emotional comfort	

Peng et al. 2021 [42], China	Insights into experiences of frontline nurses from epidemic centre	Nurses (*n* = 20)	Descriptive phenomenological research study	Semistructured face-to-face interviews Content analysis Colaizzi's Phenomenological method	(i) Fear/real risk	(i) Fear of transmission to family/friends (ii) Reflect and replan future (iii) Guilt and sadness (iv) Positive attitude
					(ii) Relationships of mutual trust	
					(iii) Media coverage	
					(iv) Nursing in isolation PPE	
					(v) Resignation	
					(vi) Pride in contribution	
					(vii) Growth in adversity	
					(viii) Dilemmas of care	

Polinard et al. 2022 [41], USA	Nurses' personal and professional experiences throughout the COVID-19 pandemic	Nurses (*n* = 45)	Qualitative research study thematic analysis	Qualitative thematic analysis of stories Inductive thematic analysis	(i) Moral identity	(i) Appreciation for family/community (ii) Self-preservation/self-care
					(ii) Connectedness to coworkers	
					(iii) Evolving policies	
					(iv) Grief experiences	
					(v) Persistence despite challenges	
					(vi) Teamwork in adversity	

Popoola et al. 2022 [51], Nigeria	An exploration of the experiences of frontline nurses caring for patients	Nurses (*n* = 15)	Descriptive research study	Semistructured Zoom interviews Template analysis	(i) Fear of contracting disease	(i) Stigmatizing and alienating (ii) Family separation (iii) Psychological well-being
					(ii) Team/interprofessional collaboration	
					(iii) Aggression transference	
					(iv) Resource constraints	
					(v) Unholistic, therapeutic time	
					(vi) Underappreciated	
					(vii) Embraced gratitude	
					(viii) Increased knowledge	

Robinson et al. 2021 [46], USA	Experiences of RNs caring for seriously ill patients with COVID‐19	Nurses (*n* = 15)	Phenomenological research study	Semistructured face-to-face interviews Transcendental phenomenology analysis	(i) Novel complexity of care	(i) Nurses in family support to nurses (ii) Resilience (iii) Sense of overwhelmed permeated personal lives
					(ii) Teamwork	
					(iii) Volume of death	
					(iv) Abandonment by leaders	
					(v) Public support/noncompliance	
					(vi) Inadequate standards	
					(vii) Role disillusionment	
					(viii) New knowledge/critical thinking	

Rodríguez-Martín et al. 2022 [47], Spain	Perceptions of nurses working frontline and caring for people with COVID-19	Nurses (*n* = 14)	Descriptive phenomenological research study	Semistructured interviews Giorgi's phenomenological method	(i) Overwhelmed with anxiety	(i) Transmission of infection (ii) Reduced socialisation (iii) Enochlophobia (fear of crowds)
					(ii) Unknown enemy	
					(iii) Questioning competencies	
					(iv) Tense work environment	
					(v) Essential teamwork	

Rony et al. 2021 [75], Bangladesh	Experiences of nurses and their perceptions in managing COVID-19 situations	Nurses (*n* = 14)	Semistructured audio-video interviews	Descriptive study Thematic analysis	(i) Resources constraints	
					(ii) Work overload	
					(iii) Change in environment	
					(iv) Substandard quality of care	
					(v) Patient empathy	

Sezgin et al. 2021 [48], Turkey	Experiences of the ICU nurses who cared for COVID-19 patients	Nurses (*n* = 10)	Descriptive research study	Semistructured Zoom interviews Thematic analysis	(i) Fear of death	(i) Fear of transmission (ii) Separation anxiety (iii) Isolation (iv) Dissatisfaction and worthlessness (v) Labelling/society/heroes
					(ii) Restriction of freedom	
					(iii) Standards of nursing care	
					(iv) Time limitation	
					(v) Substandard guidance	
					(vi) Significance of profession	
					(viii) Learning opportunity	
					(ix) Resource constraints	

Shahoei et al. 2022 [64], Iran	Exploration of nurses' experience in providing care to patients with COVID-19	Nurses (*n* = 14)	Phenomenological research study	Semistructured face-to-face interviews Data analysis Colaizzi's phenomenological method	(i) Fear of disease	(i) Fear of transmission to family (ii) Self-care
					(ii) Forced care for	
					(iii) Resource constraints	
					(iv) Protocol deficit	
					(v) Death rate	
					(vi) Altruism	
					(viii) Compassion	

Shin and Yoo 2022 [84], South Korea	The experiences among nurses in COVID-19 units	Nurses (*n* = 15)	Descriptive research study	Semistructured face-to-face interviews Content analysis	(i) Therapeutic communication	
					(ii) Core messenger	
					(iii) Personalized nursing care	
					(iv) Expansion of professionalism	
					(v) Social burden	
					(vi) Rebirth of nightingale	
					(vii) Increased self-esteem	

Specht et al. 2021 [50], Denmark	An exploration on nurses' experiences working in a newly organised COVID-19 ward	Nurses (*n* = 23)	Explorative research study	Semistructured telephone interviews Data analysis narrative and interpretation	(i) Fear of infection	
					(ii) Inadequacy/redeployment	
					(iii) Collegial solidarity	
					(iv) Novel nature	
					(v) Media-driven knowledge	
					(vi) Resource constraints	
					(vii) Undervalued role	
					(viii) Professional growth	
					(ix) Part of history	

Stayt et al. 2022 [24], UK	Exploration of nurses' experiences of patient care in intensive care during COVID-19	Nurses (*n* = 19)	Constructivist research study	Semistructured telephone interviews Thematic analysis	(i) Fear-influenced well-being	
					(ii) Value of peer support	
					(iii) Resource inadequacies	
					(iv) Professional accountability	
					(v) Professionally vulnerability	
					(vi) Suboptimal/unholistic care	
					(vii) Psychological patient care	

Taheri-Ezbarami et al. 2023 [70], Iran	Exploration of nurses' experiences of caring for patients with COVID-19	Nurses (*n* = 15)	Phenomenological research study	Semistructured interviews Directed content analysis Watson's human caring theory	(i) Uncertainty	(i) Fear for family
					(ii) Unfavourable environment	
					(iii) Values/altruism/humanistic	
					(iv) Trust in relationships	
					(v) Sensitivity to others	

Terzioglu and Kanisli 2022 [44], Turkey	Experiences of nurses caring for patients with COVID-19	Nurses (*n* = 40)	Phenomenological research study	Semistructured telephone interviews Content analysis Colaizzi's Phenomenological method	(i) Psychological fear and anxiety	(i) Loneliness and isolation (ii) Concern about childcare (iii) Separation (iv) Self-care
					(ii) Anger over noncompliance	
					(iii) Adaptation processes	
					(iv) Team cooperation	
					(v) Rapid transformation	
					(vi) Motivated by societal support	

Tinmaz and Altundag 2022 [65], Turkey	The experiences of nurses who were mothers during COVID-19	Nurses (*n* = 18)	Phenomenological research study	Semistructured face-to-face interviews Content analysis	(i) Enhanced infection control	(i) Disrupted family life
					(ii) Burnout	(ii) Separation
					(iii) Communication	(iii) Fear of transmission
					(iv) Resource constraints	(iv) Societal stigmatization
					(v) Social isolation	(v) Homeschooling affected roles

Tong et al. 2022 [85], China	Influencing factors on nurses' caring behaviour during COVID-19	Nurses (*n* = 42)	Inductive research study	Semistructured Telephone interviews Thematic analysis	(i) Caring limitations	
					(ii) Emotional regulation	
					(iii) Constraints to care	
					(iv) Public respect	
					(v) Ethical dilemma	
					(vi) Positive professional identity	
					(vii) Belief in capability	

Turgut et al. 2021 [22], Turkey	Exploration of experiences of intensive care and emergency nurses caring for COVID-19 positive patients	Nurses (*n* = 21)	Descriptive research study	Semistructured interviews Content analysis hermeneutic phenomenology	(i) Fear/threat of infection	(i) A paradox of care (ii) Fear of infecting with COVID-19 (iii) Self-care (iv) Domestic relations (v) Community stigma (vi) Exclusion
					(ii) Collegial support	
					(iii) Increased responsibility	
					(iv) Humanitarian aspect	
					(v) Media inaccuracies	
					(vi) Devalued and injustice	
					(v) Fear around death empathy	
					(vi) Positive reinforcement	
					(vii) Professional growth	
					(viii) Professional dilemmas	

Villar et al. 2021 [54], Qatar	Experiences of frontline nurses providing nursing care for COVID-19 patients	Nurses (*n* = 30)	Phenomenological research study	Semistructured face-to-face interviews Data analysis Colaizzi's phenomenological method	(i) Fear of infection	(i) Fear of transmission to family (ii) Precautionary self-imposed infection control
					(ii) Team relationships	
					(iii) Resource constraints	
					(iv) Professional pride	
					(v) Empathy with the family	

Widiasih et al. 2021 [83], Indonesia	Exploration of nurses safeguarding their families while working with COVID-19 patients	Nurses (*n* = 17)	Descriptive research study	Semistructured face-to-face interviews, face-to-face telephone, and video conferencing Content analysis		(i) Behavioural modifications
						(ii) Separation from children
						(iii) Self-care
						(vi) Family care
						(v) Preventative education for family
						(vi) Stigmatization from community

Xu et al. 2021 [55], China	The experience and views of frontline nurses in the initial stages of the COVID-19 pandemic	Nurses (*n* = 9)	Descriptive research study	Semistructured interviews conventional content analysis	(i) Fear and shame of infection	(i) Rich life experience (ii) Cohesion of whole nation (iii) Deep experience in career (iv) Positive education for children (v) Gratitude for life and family
					(ii) Inadequate resources	
					(iii) Failed life-saving	
					(iv) Unfamiliar environment	
					(v) Team transition	
					(vi) Relationship with patients	
					(vii) Performance competition	
					(viii) Death psychological	
					(ix) Nationwide recognition	
					(x) Role expansion	
					(xi) Future capability	

Zamanzadeh et al. 2021, [56] Iran	The nurses' experiences in taking care of COVID-19 patients	Nurses (*n* = 20)	Descriptive research study	Semistructured telephone interviews Content analysis	(i) Fearful of unknown	(i) Change of personal lifestyle (ii) Self-quarantine from family/friends (iii) Isolation (iv) Drop in education standards of children (v) Fear of longevity of COVID-19
					(ii) Staff empathy	
					(iii) Media coverage	
					(iv) Lack of resources	
					(v) Physical effects of PPE	
					(vi) Ineffective care	
					(vii) Counsellors for patients'	
					(viii) Community reaction	
					(ix) COVID-19 deaths	
					(x) Pride in profession	

Zhang et al. 2021 [57], China	Experiences of frontline nurses after the COVID-19 rescue	Nurses (*n* = 15)	Phenomenological research study	Semistructured face-to-face interviews Data analysis Colaizzi's phenomenological method	(i) Fear	(i) Resisting social activities
					(ii) Underperformance	
					(iii) Isolation	
					(iv) Death	
					(v) Pride-affirmed career	
					(vi) Strengthened knowledge	
					(vii) Professional confidence	
					(viii) Future crisis readiness	

**Table 4:** Application of Mesirow's framework of transformative learning theory to nurses' caring role during the COVID-19 pandemic.

Phase	Defining characteristics	Caring role in COVID-19
1	A disorienting dilemma	Caring dilemma
2	A self-examination with feelings of guilt or shame	Emotional turmoil
3	A critical assessment of epistemic, sociocultural, or psychic assumptions	Erosion of care
4	Recognition that one's discontent and the process of transformation are shared and that others have negotiated a similar change	Relationships and solidarity
5	Exploration of options for new roles, relationships, and actions	Relationships and growth
6	Planning of a course of action	Expansion of role
7	Acquisition of knowledge and skills for implementing one's plans	Professional expanded caring role
8	Provisional trying of new roles	Professional growth
9	Building of competence and self-confidence in new roles and relationships	Professional growth
10	A reintegration into one's life based on conditions dictated by one's perspective	Future capability

## Data Availability

Data supporting the findings of this scoping review study article are included within the study and/or are available from the authors upon reasonable request.

## Additional Points

*Contribution of Paper.* This paper contributes to the wider global clinical community in alignment with the United Nations Sustainable Development Goals that prioritise health and highlight that a well-supported workforce is paramount to the development of people-centred health systems, and emergency preparedness. Developing a supportive environment by listening and responding to nurses' voices is an essential element of underpinning and sustaining the profession. Adapting to changing conditions and transforming healthcare delivery demands innovative approaches, it is therefore worth investing in in transformational learning and enhanced capability of the nursing workforce, supported by further research, education and policy for inevitable future healthcare crises preparedness.
